# Ube2s stabilizes β-Catenin through K11-linked polyubiquitination to promote mesendoderm specification and colorectal cancer development

**DOI:** 10.1038/s41419-018-0451-y

**Published:** 2018-04-19

**Authors:** Zhaoyan Li, Yan Wang, Yadan Li, Wanqi Yin, Libin Mo, Xianghao Qian, Yiran Zhang, Guifen Wang, Fan Bu, Zhiling Zhang, Xiaofang Ren, Baochang Zhu, Chang Niu, Wei Xiao, Weiwei Zhang

**Affiliations:** College of Life Sciences, Capital Normal University, Beijing, China

## Abstract

The canonical Wnt/β-Catenin signaling pathway is widely involved in regulating diverse biological processes. Dysregulation of the pathway results in severe consequences, such as developmental defects and malignant cancers. Here, we identified Ube2s as a novel activator of the Wnt/β-Catenin signaling pathway. It modified β-Catenin at K19 via K11-linked polyubiquitin chain. This modification resulted in an antagonistic effect against the destruction complex/β-TrCP cascade-orchestrated β-Catenin degradation. As a result, the stability of β-Catenin was enhanced, thus promoting its cellular accumulation. Importantly, Ube2s-promoted β-Catenin accumulation partially released the dependence on exogenous molecules for the process of embryonic stem (ES) cell differentiation into mesoendoderm lineages. Moreover, we demonstrated that UBE2S plays a critical role in determining the malignancy properties of human colorectal cancer (CRC) cells in vitro and in vivo. The findings in this study extend our mechanistic understanding of the mesoendodermal cell fate commitment, and provide UBE2S as a putative target for human CRC therapy.

## Introduction

In the process of ubiquitination, ubiquitin (Ub) protein is covalently attached to substrates either as a monomer or a polymer chain linked via its N-terminus or any of its seven lysine (K) residues, K6, K11, K27, K29, K33, K48, and K63. Among them, the cellular function of K48- and K63-linked polyubiquitin chains is well understood. It is generally believed that K48 linkage marks substrates for degradation, whereas K63-linked polyubiquitination results in non-degradative outcomes, such as signal transduction^1–3^. K11-linked polyubiquitin chain is another common modification in eukaryotic cells^4,5^. Extensive studies have provided insights into its biochemical mechanisms and cellular functions in cell cycle progression, pluripotency, and differentiation^6–10^. In general, the process of ubiquitination is achieved by three types of enzymes, namely Ub-activating enzyme (Uba, E1), Ub-conjugating enzyme (Ubc, E2), and Ub ligase (E3)^3^. Ub-conjugating enzyme E2S (Ube2s) is a K11 linkage-specific E2^11,12^. It selectively cooperates with E1, another priming E2 (Ube2c/d), and the E3 complex anaphase-promoting complex/cyclosome (APC/C) to elongate K11-linked polyubiquitin chain on substrates for 26S proteasome-mediated degradation^6,8,10^. The critical role of Ube2s in regulating cell cycle and differentiation inevitably implicates it into tumorigenesis. To date, aberrant expression of Ube2s has been detected in multiple human primary cancers^13–15^. Strikingly, Ube2s overexpression alone is sufficient for the onset of some types of cancers^15^.

The canonical Wnt/β-Catenin signaling pathway pivotally regulates diverse cellular processes, including embryonic development, stem cell maintenance, and differentiation^16,17^. As the core component of this pathway, β-Catenin is tightly regulated by post-translational modifications that fine-tune its protein level and optimal activity. At the molecular level, when Wnt ligands bind to the Frizzled receptor and its co-receptor, low-density-lipoprotein-related protein 5/6 (LRP5/6), β-Catenin is dissociated from the Axin destructive complex and subsequently translocates from the cytoplasm into nucleus for transcription regulation^18^. The Axin destructive complex is composed of several proteins, including Axin, glycogen synthase kinase 3 (GSK3), adenomatous polyposis coli (APC), and casein kinase 1 (CK1). In the absence of activation stimuli, β-Catenin is recruited to the destructive complex for sequential phosphorylation at serine 45 (S45) by CK1 followed by S33, S37, and threonine 41 (T41) by GSK3^19–21^. Consequently, the phosphorylated S33 and S37 of β-Catenin act as the signals recognized by an E3 complex Skp1/Cul1/F-box^β-TrCP^ which promotes K48-linked polyubiqutination and proteasomal degradation ^18,22–26^.

Interestingly, several lines of evidence suggest the potential association between Ube2s and β-Catenin. Previous studies reported that transcription factor SRY (sex-determining region Y)-box 2 (Sox2) is an interaction partner of β-Catenin in breast cancer and mouse embryonic stem (mES) cells ^10,27^. Meanwhile, Sox2 is associated with Ube2s via direct physical interaction^10^. Sharing a common interacting partner suggests that Ube2s and β-Catenin may be functionally connected in the same pathway. In addition, *Ube2s*-overexpressed mES cells exhibit specific induction of *Esrrb* which is a downstream target of the Wnt/β-Catenin signaling^10,28^, indicating that Ube2s could serve as an activator of the pathway. Importantly, β-Catenin has been found to be modified by K11-linked polyubiquitin chain^29^. Since Ube2s is one of the most established E2 mediating K11 linkage, it could potentially be involved in monitoring the cellular activity of β-Catenin. In this study, we explored the role of Ube2s in regulating β-Catenin and uncovered that Ube2s directly interacted with β-Catenin to ubiquitinate its K19 residue via K11 linkage. This modification promoted β-Catenin stablization through antigonizing its proteasomal degradation mediated by the destruction complex/β-TrCP signaling. Consequently, Ube2s promoted mesoendoderm lineage specification from mES cells. Meanwhile, it enhanced the malignancy properties of colorectal cancer (CRC) both in vitro and in vivo, which can be markedly reduced upon *UBE2S* deletion alone. Our study presents UBE2S as a potential novel target for enhanced CRC treatments and production of specific mesoendodermal lineages from mES cells.

## Results

### Ube2s interacted with β-Catenin to enhance its protein stability

**Fig. 1:**
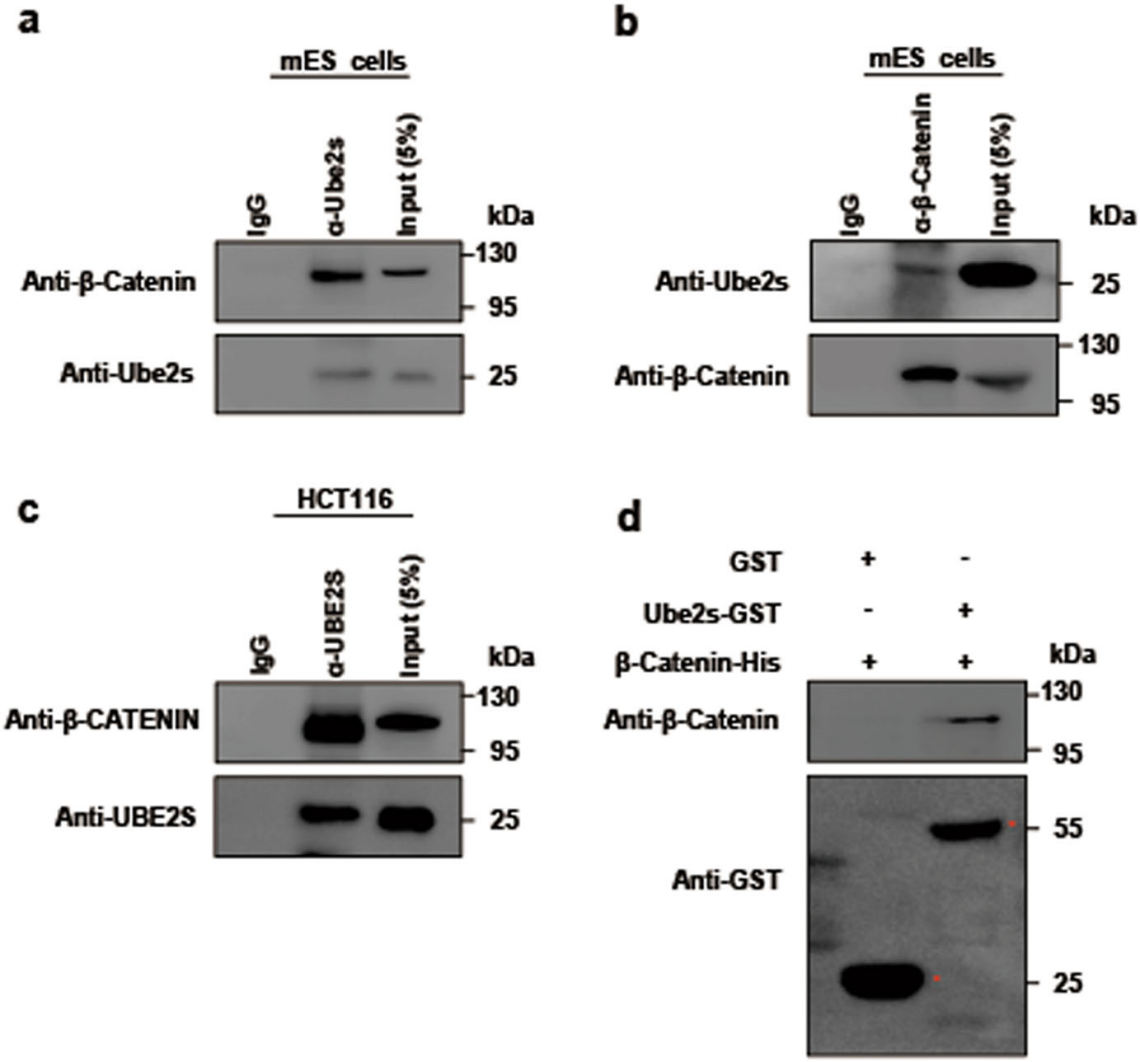
Ube2s interacted with β-Catenin. **a**, **b** co-IP to detect the association of Ube2s with β-Catenin in mES cells. Whole cell extracts of mES cells were subjected to co-IP with the antibody against Ube2s (**a**) or β-Catenin (**b**), followed by western blotting with antibodies against β-Catenin and Ube2s, respectively. **c** co-IP to detect the interaction between UBE2S and β-CATENIN in HCT116 cells. **d** A pull-down assay to analyze the direct association of Ube2s with β-Catenin. Purified GST-tagged Ube2s was conjugated to glutathione–sepharose beads, and subsequently incubated with purified His-tagged β-Catenin. The elution was analyzed by western blot using the anti-β-Catenin and anti-GST antibodies, respectively. GST alone was used as a control. The data presented are based on three independent repeats

We first checked the interaction between Ube2s and β-Catenin. Whole cell extracts of mES cells were isolated for interrogation by a co-immunoprecipitation (co-IP) assay. Results confirmed their endogenous interaction (Fig. 1a, b). Moreover, this association can be observed across other cell types, such as CRC HCT116 cells, pluripotent F9 teratoma cells, and human embryonic kidney 293 cells (Fig. 1c and Supplementary Figure S1a-b). Next, we obtained the cytoplasm and nuclear extracts of mES cells, respectively, for the co-IP assay. Results showed that the Ube2s–β-Catenin complex can be detected with both subcellular fractionations (Supplementary Figure S1c). Furthermore, we purified recombinant Glutathione S-transferase (GST)-tagged Ube2s and His_6_-tagged β-Catenin fusion proteins to interrogate their association using an in vitro GST pull-down assay. Our result showed that they were capable of forming a protein complex through direct physical interaction (Fig. 1d).

**Fig. 2:**
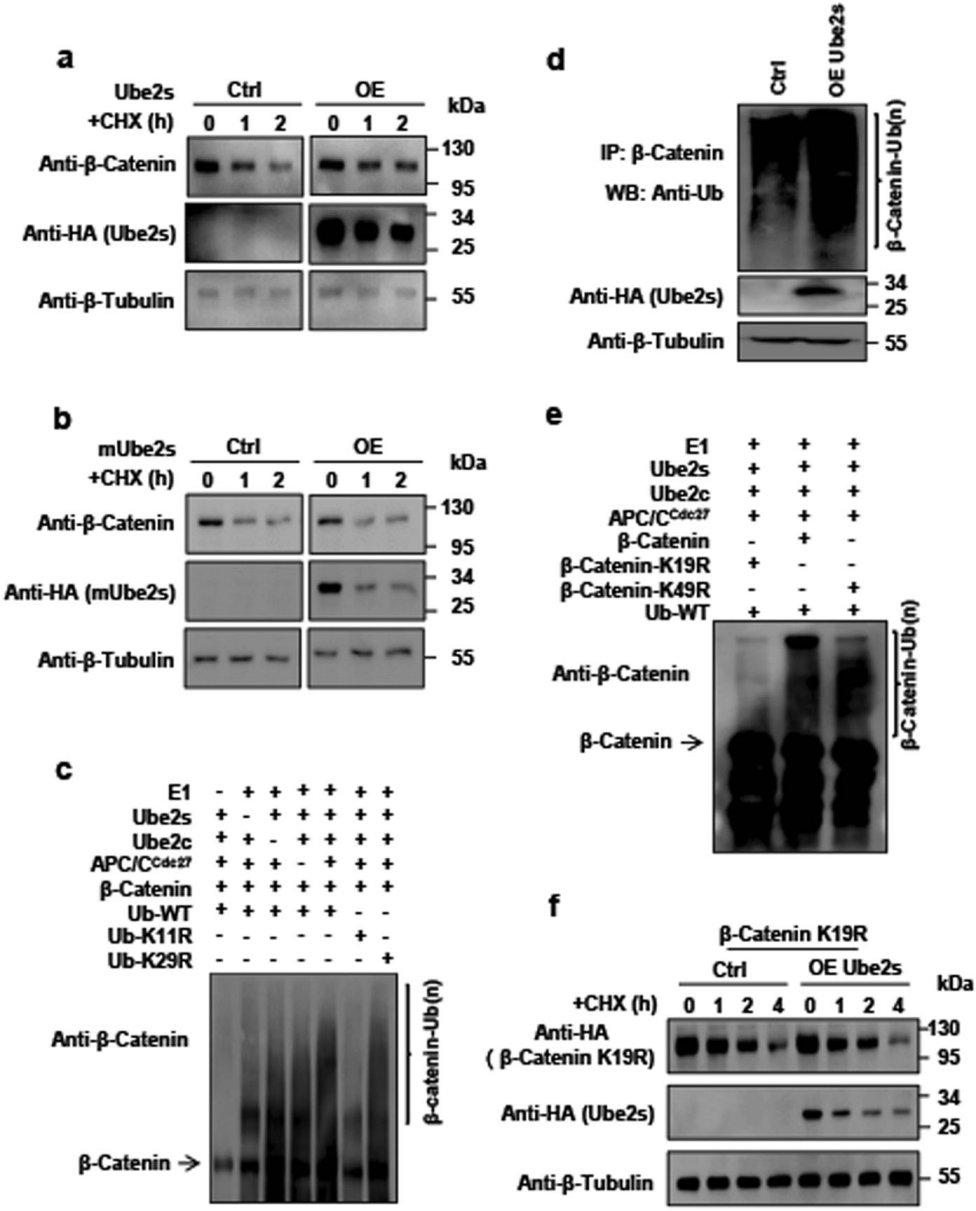
Ube2s ubiquitinated β-Catenin to enhance its stability. **a** *Ube2s* overexpression inhibited β-Catenin degradation. The construct overexpressing *Ube2s* was transfected into mES cells. The cells were treated with 12.5 µg/ml CHX for the indicated hours. **b** Ube2s-C95S mutant failed in stabilizing β-Catenin. mES cells were transfected with the construct expressing Ube2s-C95S. Experimental design and procedures are as described in **a**. Ctrl: vector transfection control; OE: overexpression. **c** Ube2s-mediated β-Catenin ubiquitination in vitro. The reaction components for in vitro ubiquitination assay include purified His_6_-tagged Ub, the E3 APC^Cdc27^ complex co-IP by Cdc27 from mES cells, and purified E1 (Uba1), Ube2c, Ube2s, and β-Catenin proteins. The reaction solution was analyzed by western blot with an anti-β-Catenin antibody. **d** Ube2s promoted β-Catenin ubiquitination in vivo in mES cells. mES cells were transfected with a construct expressing Ube2s; 3 µM MG132 was added to treat cells for 15 h. Whole cell proteins were prepared for co-IP analysis with an anti-β-Catenin antibody, followed by western blotting with an anti-Ub antibody. **e** In vitro ubiquitination assay to identify β-Catenin-K19 as the ubiquitination site by Ube2s. Wild-type β-Catenin, β-Catenin-K19R, and β-Catenin-K49R were purified, respectively, for the in vitro ubiquitination assay. **f** Ube2s failed in stabilizing β-Catenin-K19R. The construct overexpressing *Ube2s* and empty vector were respectively co-transfected with the plasmid expressing β-Catenin-K19R into mES cells. Similar experimental procedure was employed with **a**. The data presented are based on three independent repeats

The canonical function of Ube2s is to promote degradation of substrates via ubiquitination. Thus, we sought to determine whether Ube2s participated in regulating the stability of β-Catenin. We exogenously manipulated the level of Ube2s through its overexpression in mES cells. When combined with cycloheximide (CHX) treatment to block de novo protein biosynthesis, we found that Ube2s elevation failed to promote the degradation of β-Catenin. Instead, it enhanced the β-Catenin stability (Fig. 2a). Furthermore, overexpression of enzymatically inactive Ube2s-C95S mutant^6^ failed to retard the degradation of β-Catenin (Fig. 2b). This observation demonstrated that the regulation of β-Catenin stability via Ube2s was dependent on its E2 enzymatic activity. As *Ube2s* depletion results in mES cell differentiation, which in turn affects the expression level of β-Catenin^10,30–32^, we could not conclusively test the effect of *Ube2s* depletion on β-Catenin stability although we observed a reduction in β-Catenin in *Ube2s-*depleted mES cells (Supplementary Figure S2a). Next, we checked the effect on HCT116 cells. Results showed that the overall level of β-CATENIN was decreased upon *UBE2S* depletion while increased with UBE2S overexpression (Supplementary Figure S2b-c). Consistently, UBE2S elevation-induced β-CATENIN accumulation was observed in another CRC cell line HT29 (Supplementary Figure S2d). The mRNA level of *β-Catenin* did not exhibit any significant change upon Ube2s manipulation (Supplementary Figure S2e). Collectively, we conclude that Ube2s stabilizes β-Catenin and enhances its cellular accumulation. It is generally believed that Ube2s-mediated K11 polyubiquitin linkage marks substrates for degradation^8,10^. However, this notion is severely challenged by our findings. Previous studies provided indications that K11 linkage may indeed increase the stability of β-Catenin in cancer cells^29^. Of note, functional diversity of polyubiquitin chains is not restricted to the K11-linked signal. For example, K63-linked polyubiquitination promotes 26S proteasome-mediated substrate proteolysis, but has additional function as a scaffold for protein assembly in signaling pathways ^33–35^.

### Ube2s ubiquitinated β-Catenin at residue K19

We asked if Ube2s directly modifies β-Catenin via ubiquitination. First of all, we examined the association of β-Catenin with the Cdc27 subunit of APC/C, which is required for Ube2s-mediated polyubiquitination^36^. Co-IP assay revealed that these two proteins indeed interacted in the mES cells (Supplementary Figure S3a). Next, we performed an in vitro ubiquitination assay and found that Ube2s successfully assembled polyubiquitin chains on β-Catenin (Fig. 2c). In addition, the polyubiquitin signal was dramatically reduced with the introduction of Ub-K11R mutant, highly suggestive that Ube2s mediates K11 linkage formation on β-Catenin (Fig. 2c). The study by Hay-Koren et al. shows that stability of β-Catenin could be increased with K11- or K29-linked polyubiquitination^29^. We checked whether Ube2s mediated K29-linked polyubiqutination of β-Catenin. As shown in Fig. 2c, mutation in the K29 residue of Ub failed in compromising Ube2s-mediated polyubiquitination, suggesting that Ube2s indeed modifies β-Catenin specifically via K11 linkage, and not K29. Furthermore, we checked Ube2s-catalyzed polyubiquitination of β-Catenin in vivo. The cell lysates of *Ube2s*-overexpressed mES and HCT116 cells were prepared for the co-IP assay. Results showed that elevation in Ube2s dramatically increased the level of polyubiquitinated β-Catenin in both cell types (Fig. 2d, Supplementary Figure S3b). Collectively, we conclude that Ube2s is capable of modifying β-Catenin with K11-linked polyubiquitin chain both in vitro and in vivo.

Next, we wished to define the exact residue of β-Catenin ubiquitinated by Ube2s. Previous studies reveal that phosphorylated β-Catenin can be recognized and modified by the Ub ligase β-TrCP at K19 and K49 residues for degradation^25,37–39^. We hypothesized that Ube2s-APC/C complex competed with β-TrCP to ubiquitinate the K19 and/or K49 residue(s) of β-Catenin to prevent its degradation. To this end, we expressed β-Catenin mutants in which the K19 and K49 residues were substituted with an arginine (R), respectively. Using in vitro ubiquitination assay, we found that the K19R mutation compromised the Ube2s-mediated polyubiquitin signal on β-Catenin, while the K49R mutation did not display any effect (Fig. 2e). Consistently, Ube2s failed in stabilizing β-Catenin-K19R mutant (Fig. 2f). These findings demonstrate that Ube2s facilitates ubiquitination of β-Catenin via the K19 residue to increase its stability.

### Ube2s-mediated ubiquitination antigonized the degradation signals on β-Catenin

**Fig. 3:**
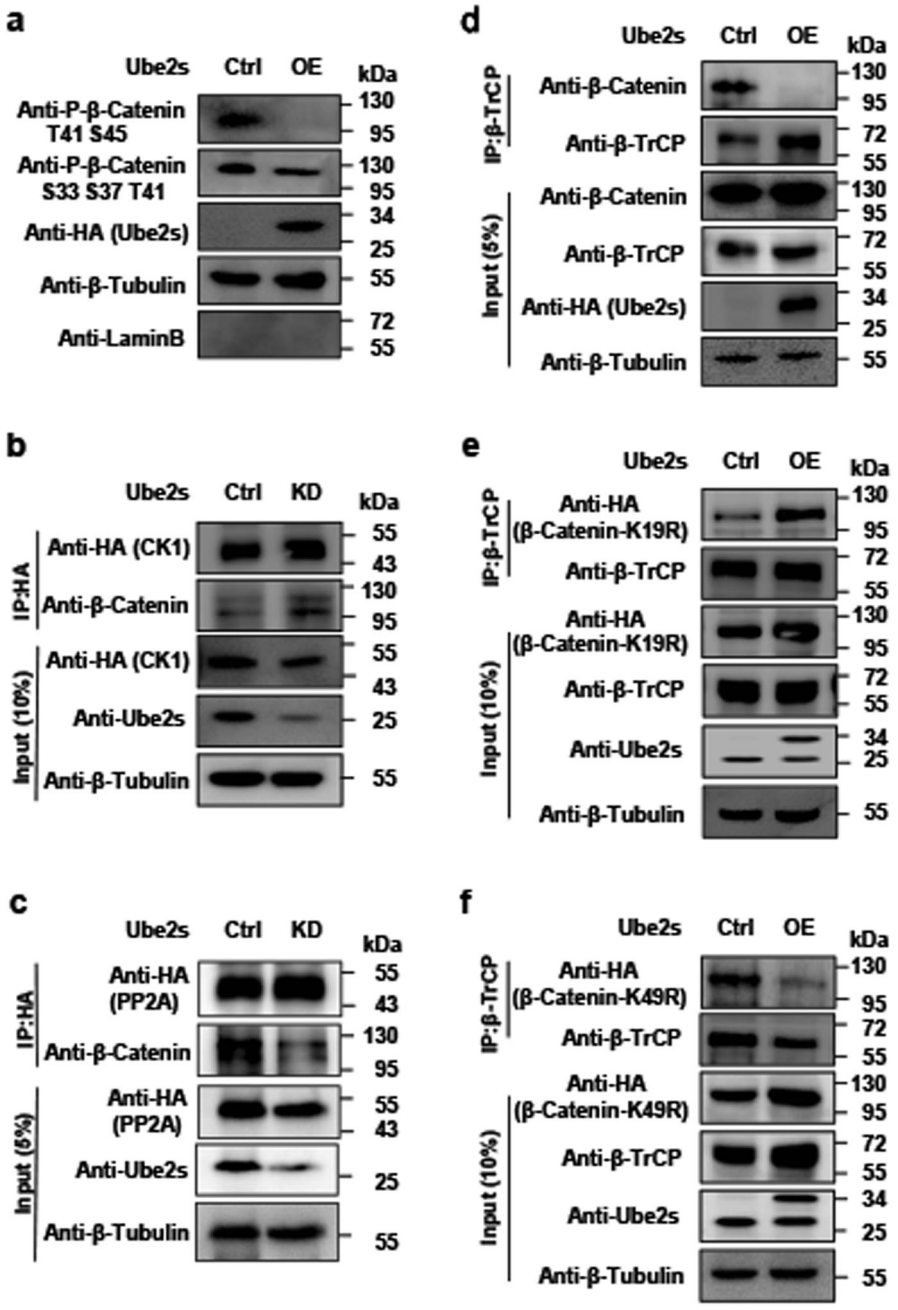
Ube2s allowed β-Catenin to escape from the destruction complex-mediated degradation. **a** Ube2s elevation downregulated repressive phospho-β-Catenin in mES cells. The construct overexpressing HA-tagged Ube2s was transfected into mES cells. After 2-day antibiotic selection, cytoplasm extracts were prepared for western blotting assay with the antibodies against phospho-β-Catenin at T41/S45 and S33/S37/T41, respectively. The antibodies against Tubulin and LaminB were used as markers for cytoplasm and nuclear, respectively. **b** *Ube2s* depletion promoted the interaction between β-Catenin and CK1. The constructs expressing Ube2s shRNA and HA-tagged CK1 were co-transfected into mES cells for the co-IP assay. **c** *Ube2s* depletion repressed the interaction between β-Catenin and PP2A. Similar experimental strategy with **b** was employed. **d** Ube2s repressed the interaction between β-Catenin and β-TrCP. Whole cell extracts of the mES cells overexpressing HA-tagged Ube2s were obtained for the co-IP assay with the antibody against β-TrCP, followed by western blotting with antibodies against β-Catenin and β-TrCP, respectively. **e** Ube2s failed in inhibiting the interaction of β-TrCP with β-Catenin-K19R mutant. Transfect the plasmid inserted with Ube2s cDNA and the empty vector (Ctrl), respectively, into mES cells expressing β-Catenin-K19R mutant. Two days after transfection, whole cell extracts were obtained for the co-IP assay. **f** Ube2s inhibited the interaction of β-TrCP with β-Catenin-K49R mutant. Similar experiment was performed with **e**. All data are based on three independent repeats

Several studies reveal that phosphorylation of β-Catenin at S33/S37/S45/T41 is the hallmark of β-TrCP-promoted degradation^19,37,38,40–42^. Therefore, we hypothesized that Ube2s-mediated polyubiquitination could deprive these repressive phosphorylation signals and subsequently shield β-Catenin from its recognition by β-TrCP. To test this notion, we elevated the expression level of Ube2s in mES cells. The cytoplasm extracts were prepared and subjected to a western blotting assay with antibodies against repressive phospho-β-Catenin. Results showed that Ube2s elevation indeed decreased the levels of phosphorylated β-Catenin at residues S33/S37/T41/S45 (Fig. 3a). Similar results were obtained in HCT116 cells (Supplementary Figure S3c). Since CK1 mediates β-Catenin phosphorylation that can be directly removed by Protein Phosphatase 2A (PP2A)^24^, we asked whether Ube2s regulated the interaction of β-Catenin with these two factors. *Ube2s*-depleted cell lysates were prepared and subjected to the co-IP assay. As shown in Fig. 3b, c, the protein levels of CK1 and PP2A were maintained. However, the CK1/β-Catenin complex formation was induced, while the PP2A/β-Catenin complex was largely removed. This observation explains Ube2s-mediated inhibition of β-Catenin phosphorylation. Since β-TrCP-mediated β-Catenin degradation depends on the phosphorylation signals, their downregulation could directly inhibit the recognition of β-Catenin by β-TrCP. As expected, Ube2s elevation abolished the interaction between β-Catenin and β-TrCP (Fig. 3d). Furthermore, this effect was retarded by K19R mutation in β-Catenin, rather than K49R (Fig. 3e, f). These observations demonstrate that Ube2s represses β-TrCP-mediated degradation of β-Catenin specifically through its ubiquitination at the K19 residue.

### Ube2s promoted mES cell differentiation into mesoendoderm

**Fig. 4:**
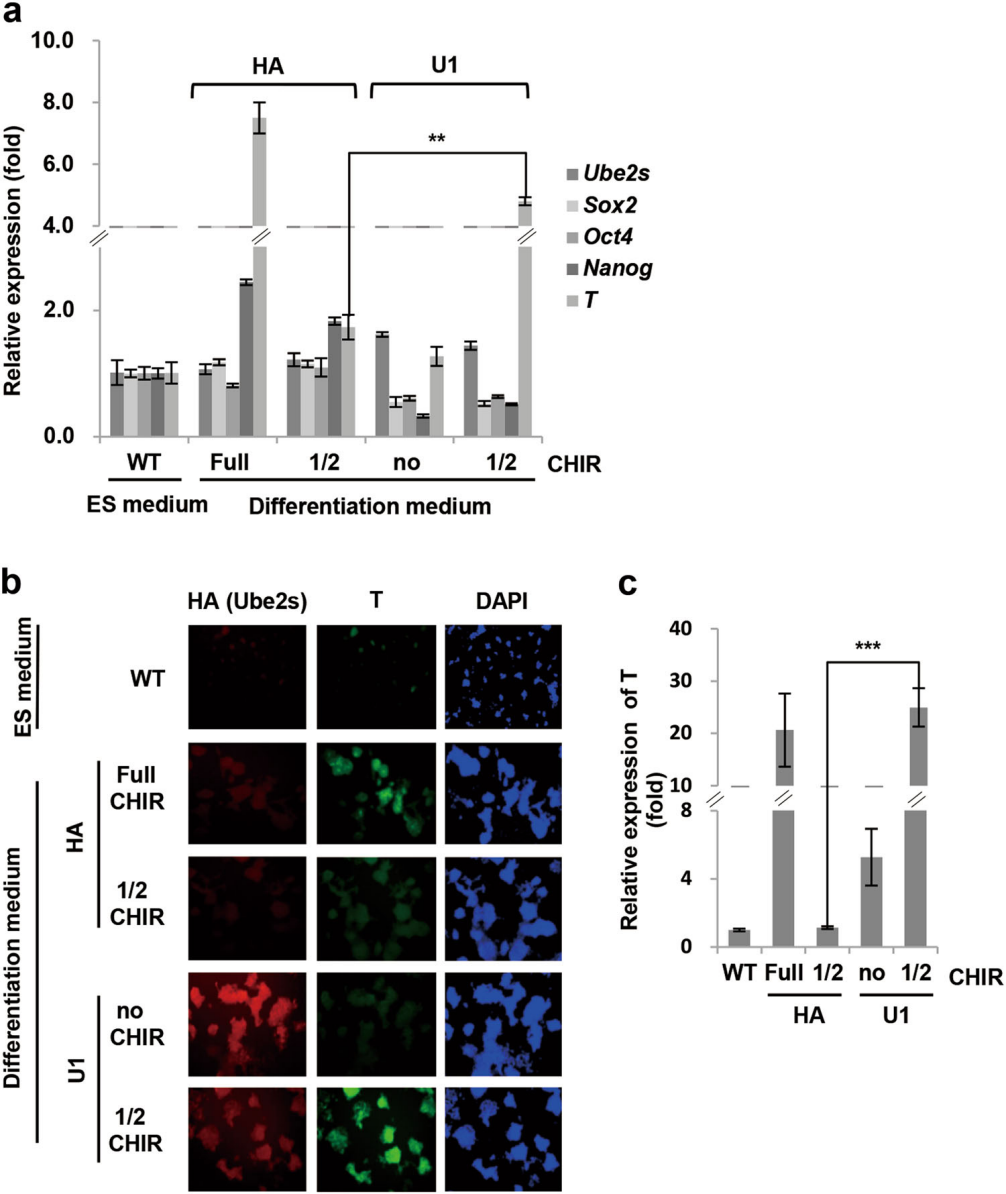
Ube2s promoted mES cell differentiation into mesoendoderm. **a**, **b** Ube2s overexpression stable mES cell line U1 and the vector-transfected control line (HA) were induced to differentiate into mesoendoderm. The resulting cells were analyzed by qRT-PCR (**a**; *t*-test, **p < 0.01) and immunocytochemistry staining assay with antibodies against HA (Ube2s), T and DAPI (**b**). Undifferentiated mES cells (wild-type, WT) were used as controls. Photographs were taken with 200× magnification. **c** The mean DAPI-normalized staining signals of T from **b** were quantified by NIH Image J software. The values represent ratios against WT (lower panel; *t*-test; ***p < 0.001). All data presented are based on three independent repeats

With defined culture medium for differentiation, activation of the canonical Wnt/β-Catenin signaling cascade by GSK3 inhibitor CHIR99021 directs mES cells differentiation into mesoendoderm lineages^43^. Since Ube2s stabilizes β-Catenin, it may be capable of promoting this differentiation process. To test the possibility, we checked whether Ube2s-promoted β-Catenin accumulation could release the dependence on exogenous molecule CHIR99021 for the process of in vitro mesoendoderm differentiation from mES cells. We established two independent mES cell lines (U1 and U2) stably overexpressing Ube2s. Realtime PCR and ICC results showed that with 3 μM CHIR99021 treatment in the defined culture medium, the control cells (HA, vector transfection) displayed a dramatic increase in mesoendoderm marker T, implicating a successful induction of differentiation (Fig. 4a–c). As expected, when the dosage of CHIR99021 was reduced by two-fold (1.5 μM), the induction in T was abolished in differentiated HA cells. However, both U1 and U2 cell lines maintained high expression levels of T, although they could not resist a complete removal of CHIR99021 (Fig. 4a–c; Supplementary Figure S4). Hence, Ube2s is able to promote mesendoderm specification.

### Ube2s enhanced the capability of CRC cells in proliferation and metastasis

**Fig. 5:**
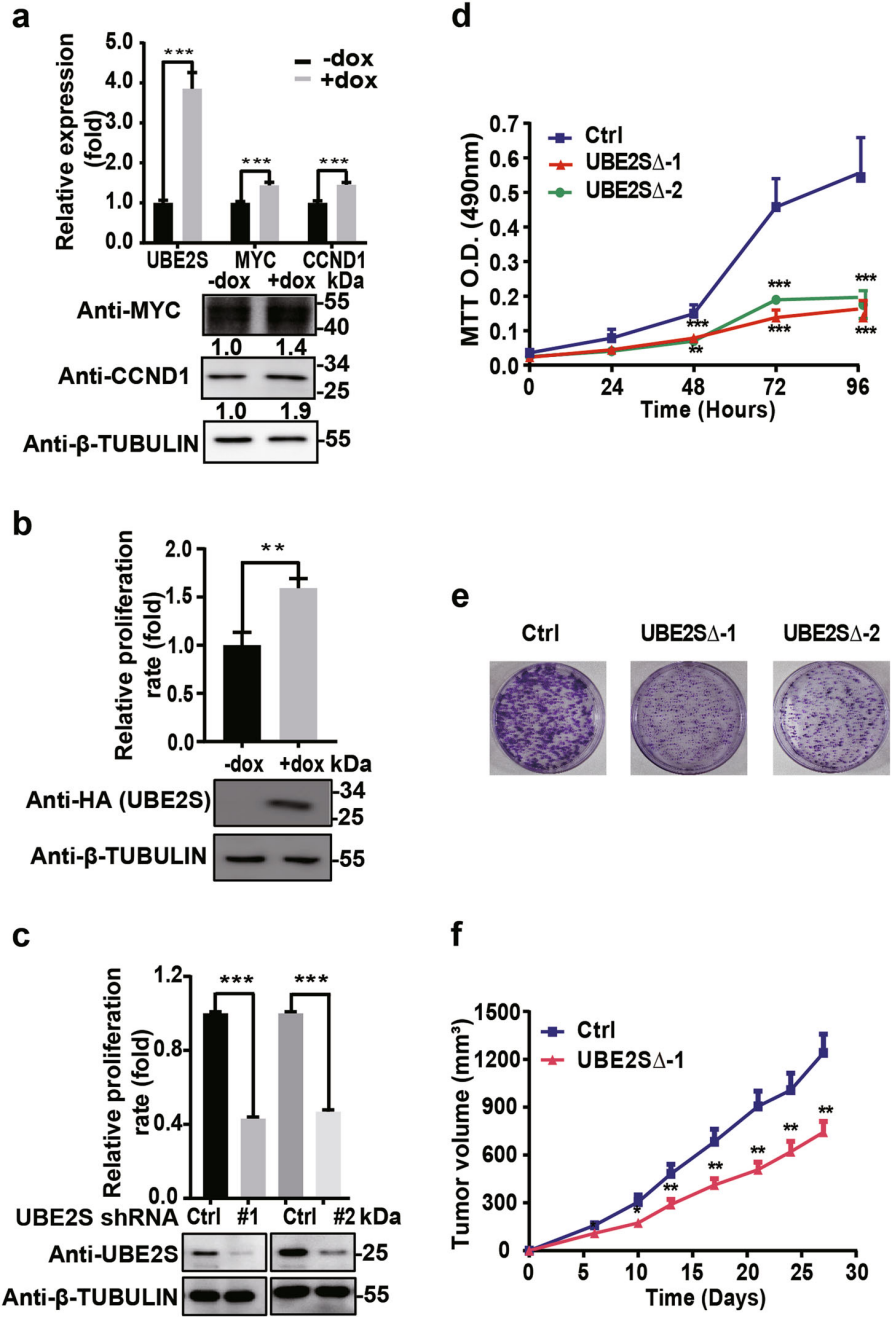
UBE2S enhanced colorectal cancer development. **a** Dox-induced UBE2S elevation activated the downstream targets of β-CATENIN. Relevant band intensities from western blot were measured by MultiGauge software (Fujifilm), and numbers were further determined by normalizing β-TUBULIN band intensity to MYC and CCND1 band intensity, respectively. **b** UBE2S elevation promoted proliferation of colorectal cancer cells. HCT116 cells expressing dox-inducible UBE2S were seeded into 6-well plate. The cell numbers were counted on the third day after dox treatment. The untreated cells HCT116 cells were used as controls. The level of ectopic UBE2S expression was monitored by western blot with the antibody against HA. **c** *UBE2S* knockdown inhibited proliferation of colorectal cancer cells. Two constructs expressing *UBE2S* shRNAs were transfected into HCT116 cells, respectively. The cell numbers were counted on the fourth day after transfection. Western blot with the antibody against UBE2S monitored the level of Ube2s. **d** The MTT assay was used to measure the proliferation of the *UBE2S*∆ HCT116 cells. **e** The colony formation assay was used to measure cell proliferation. The cells were stained with crystal violet (0.5% in 20% ethanol) for 10 min. **f** Xenograft mouse models quantitatively examined the tumor growth at multiple time points after injection of *UBE2S*∆−1 and control cells; *t*-test: ***p < 0.001; **p < 0.01; *p < 0.05. The data presented in **a**–**e** are based on three independent repeats. The number of mice for each group in **f** is seven

Previous studies have documented the gatekeeping role of β-CATENIN in promoting CRC development^44–48^. We checked whether UBE2S contributed to the malignant properties of CRC cells. We established a HCT116 cell line stably expressing doxycycline (dox)-inducible UBE2S. The downstream targets of β-CATENIN, *MYC* and *CCND1*, were activated upon dox induction (Fig. 5a). Meanwhile, the growth rate of HCT116 cells was significantly increased by dox-induced UBE2S overexpression (Fig. 5b). Conversely, HCT116 growth was reduced by short hairpin RNA (shRNA)-mediated *UBE2S* depletion (Fig. 5c). At the same time, we established *UBE2S*-deleted HCT116 cells (UBE2S∆) using the CRISPR/Cas9-mediated gene knockout system. Two individual colonies (UBE2S∆-1 and UBE2S∆-2) were prepared for further characterization (Supplementary Figure S5a-c). Consistently, phosphorylated β-CATENIN was induced and β-CATENIN target *CCND1* was repressed by *UBE2S* deletion (Supplementary Figure S5d-e). Strikingly, in these cells, the oncogenic defect in β-CATENIN degradation was corrected and β-CATENIN was conferred sensitivity to cellular degradation machinery, implying a reduced malignancy outcome (Supplementary Figure S5f). Consistently, both the MTT (3-(4,5-dimethylthiazol-2-yl)-2,5-diphenyltetrazolium bromide) assay and the colony formation assay showed that CRC cell proliferation was markedly decreased (Fig. 5d, e). Next, the *UBE2S*∆-1 and the control cells were injected subcutaneously into the bilateral flanks of nude mice. The diameters of tumors were recorded at multiple time points. Compared with the control group, the *UBE2S*∆-1 cells exhibited a significant decrease in growth (Fig. 5f).

**Fig. 6:**
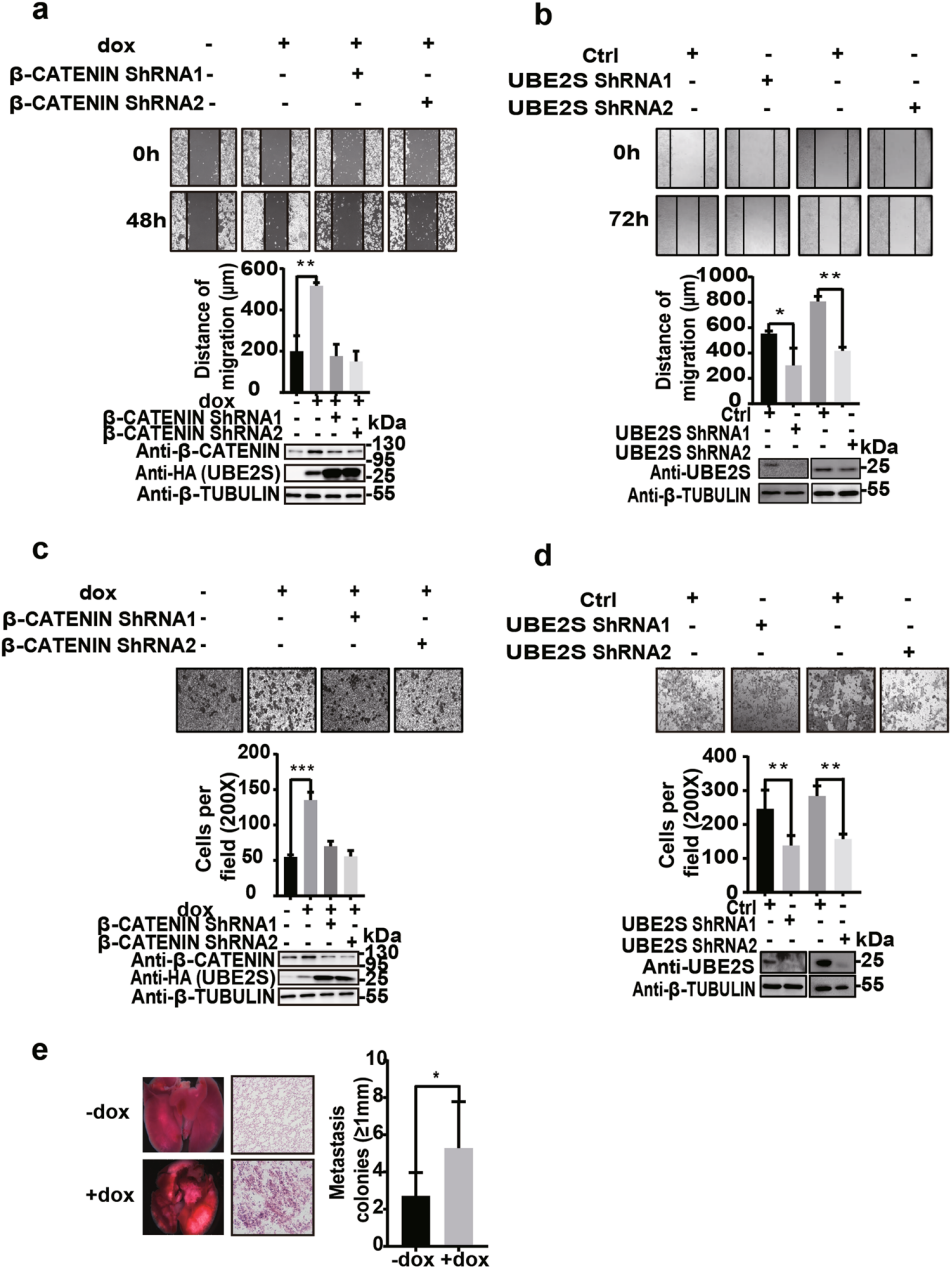
UBE2S enhanced the ability of colorectal cancer cells in migration and invasion in vitro and in vivo. **a** The scratch assay monitored the migration capacity with the HCT116 cells expressing exogenous UBE2S and *β-CATENIN* shRNAs. The dox-untreated and dox-treated HCT116 cells without *β-CATENIN* depletion were used as controls. **b** The scratch assay examined the effect of *UBE2S* depletion on HCT116 cell migration. **c** The cells of **a** were examined by the transwell assay. Five fields were randomly picked to count the cells which invade the lower surface of the filter for statistical analysis. **d** The transwell assay examined the effect of *UBE2S* depletion on HCT116 cell invasiveness. Similar statistical analysis with **c**. **e** UBE2S promotes lung metastasis of CRC cells. The left panel: representative images of lungs isolated from the nude mice. The paraffin-embedded lung tissues were analyzed by H&E staining; the right panel: the number of metastasis colonies (≥1 mm in diameter) per lung were counted; *t*-test: ***p < 0.001; **p < 0.01; *p < 0.05. The data presented in **a**–**d** are based on three independent repeats. The number of mice for each group in **e** is seven

Next, we investigated the putative role of UBE2S in regulating metastasis of CRC cells. The scratch and transwell assays were employed to assess cell migration and invasion, which are suggestive of metastasis. Results obtained by the scratch assay showed that UBE2S overexpression increased cell mobility on the second day of dox induction (Fig. 6a). Consistently, RNAi-mediated *UBE2S* depletion significantly compromised the mobility of the CRC cells (Fig. 6b). On the other hand, using the transwell assay, we observed that UBE2S elevation induced the cell invasiveness as compared with the control, whereas *UBE2S* depletion markedly decreased the capacity of HCT116 cells in terms of invasiveness (Fig. 6c, d). More importantly, RNAi-mediated *β-CATENIN* knockdown abrogated UBE2S elevation-enhanced cell mobility and invasiveness, suggesting that β-CATENIN acts as the downstream effector to mediating UBE2S-promoted CRC metastasis (Figs. 6a, c). To further determine the capacity of UBE2S in promoting CRC metastasis in vivo, we injected the dox-inducible cells into tail veins of nude mice and examined lung metastasis. Results showed that *UBES2S* overexpression markedly enhanced lung metastasis of CRC cells (Fig. 6e).

Combining our findings, we conclude that UBE2S serves as a key regulator to promote growth and metastasis of CRCs.

## Discussion

Dysregulation of the Wnt/β-Catenin pathway frequently results in severe consequences, such as malignant CRCs^49–54^. To date, however, it is still challenging to develop effective molecules that specifically target this pathway for improved cancer therapy. Most CRCs display mutations either in the APC gene or β-Catenin loci targeted by GSK3 for phosphorylation^48,51,55^. Both mutations allow β-Catenin to escape degradation and aberrantly accumulate, which has been defined as the earliest event in the process of colorectal tumorigenesis. Therefore, in order to effectively remove the accumulated β-Catenin, one potential avenue is to inhibit its stabilizing factors. Of note, several ubiquitination-related factors have been identified for their ability in stabilizing β-Catenin in different cell types. In detail, E2 Rad6B mediates K63-linked polyubiquitination of β-Catenin at residue K394 to increase its stability in breast cancer cells^56,57^. Another study identified E3 ligase EDD that modifies β-Catenin with K29- or K11-linked polyubiquitin chain to enhance its cellular accumulation^29^. The study by Dao and colleagues found that E3 FANCL modifies β-Catenin at K residue(s) rather than K19 or 49 with K11- or K63-linked polyubiquitin chain to increase its accumulation and activity in hematopoietic stem cells^58^. Although all these factors stabilize β-Catenin, their activity exhibits cell-type specificity. Accordingly, repression of these factors in CRC cells may not realize an effective removal of the redundant β-Catenin. For instance, *EDD* depletion fails in decreasing β-Catenin in CRCs, which rules out the possibility of its involvement in regulating CRC pathogenesis^29^. In this study, we identify Ube2s as a novel post-translational modifier of β-Catenin. It ubiquitinates β-Catenin via K11 linkage at its residue K19 and thus increase the stability of β-Catenin. Strikingly, manipulating the expression level of Ube2s alone appears to be sufficient for controlling the proliferation and metastasis of CRC cells. As such, our study strongly suggests Ube2s as a putative target for a future therapy of CRCs.

Besides tumorigenesis, the Wnt/β-Catenin pathway is critically involved in regulating pluripotency and cell differentiation^30–32^. For example, CHIR99021 coordinates with LIF and/or PD0325901 to maintain the self-renewal and pluripotency of mES cells in serum-free culture medium^59^. At the molecular level, CHIR99021 enhances cytosolic β-Catenin accumulation and subsequent nuclear translocation to activate Esrrb which helps to maintain the stemness of ES cells^28^. However, without LIF or PD0325901, CHIR99021 alone fails to maintain the undifferentiated state of mES cells, instead it promotes differentiation^59^. Furthermore, with defined culture medium in vitro, CHIR99021 treatment-induced β-Catenin accumulation can specifically direct mES cell differentiation into mesoendoderm lineages^43^. Two other studies also provide evidences showing that sustained activation of Wnt/β-Catenin pathway stimulates mesoendoderm lineage formation from ES cells^60,61^. By virtue of its control of β-Catenin stability, Ube2s asserts an important role in the differentiation process. Of note, our previous study shows that Ube2s ubiquitinates Sox2 and promotes its proteasomal degradation. Interestingly, the study by Thomson et al. shows that Sox2 inhibits mesoendoderm lineage formation^43^. Therefore, Ube2s exhibits twofold effect in promoting mesoendoderm differentiation from mES cells through enhancing β-Catenin accumulation and simultaneously removing the inhibitory Sox2. As such, engineering the level of Ube2s could serve as a key step toward the optimization of the protocols for in vitro mesendoderm lineage differentiation. It is noteworthy that β-Catenin can function independent of the Wnt signaling pathway. It associates with multiple Cadherin proteins, such as E-Cadherin, to regulate cell–cell adherence^62,63^. Interestingly, E-Cadherin-related intercellular adherence is required by ES cell maintenance and differentiation^64^. Thus, we could not rule out the possibility that Ube2s regulates mesoendodermal lineage commitment through its ubiquitination of E-Cadherin-linked β-Catenin.

## Materials and methods

### Cell culture

Mouse E14 ES cells (ATCC) were cultured under a feeder-free condition at 37 °C with 5% CO_2_. The cells were maintained on gelatin-coated dishes in Dulbecco’s modified Eagle medium (DMEM; GIBCO), supplemented with 15% heat-inactivated fetal bovine serum (FBS; GIBCO), 0.1 mM β-mercaptoethanol (GIBCO), 2 mM L-glutamine, 0.1 mM MEM non-essential amino acid, 5000 units/ml penicillin/streptomycin, and 1000 units/ml of LIF (ESGRO, ESG1107). Mouse F9 teratoma cells, human embryonic kidney 293 cells and human CRC HCT116 and HT29 cells were obtained from ATCC (Manassan, VA, USA). The cells were cultured in DMEM supplemented with 10% FBS, 100 units/ml penicillin, and 100 μg/ml streptomycin (Invitrogen) at 37 °C with 5% CO_2_.

### Plasmids and cell transfection

Mouse *Ube2s, β-Catenin-K19R*, and *β-Catenin-K49R* ORFs were inserted into vector pCAG-puro, respectively, for overexpression in mES cells. Human *UBE2S* ORF was inserted into vector pcDNA4.0/TO(+) for overexpression in HCT116 cells. The *Ube2s* shRNAs were cloned into pSuperpuro (Oligoengine, Seattle, WA, USA) between BglII and HindIII sites, and 19-bp shRNAs with a 9-bp loop were expressed by the pSuperpuro plasmids. The two target sequences for Ube2s knockdown in mES cells are 5′-GCTACTTCCTGACTAAAAT-3′ and 5′-GGAGGTCTGTTCCGTATGA-3′. The two target sequences for Ube2s knockdown in HCT116 cells are 5′-GCATCAAGGTCTTTCCCAA-3′ and 5′-GGCGGTTATAAAGAGGCAG-3′. Transfection of plasmids was performed using Lipofectamine 2000 (Invitrogen, 11668-019).

### Protein purification

The recombinant GST-tagged proteins were expressed in BL21 after induction with 0.2 mM IPTG at 16 °C and conjugated to glutathione–sepharose beads (GE) in lysis buffer (50 mM Tris pH 8.0, 10% glycerol, 0.3 M NaCl, 2 mM EDTA, 0.1% Triton X-100, 3 mM DTT, 1 mM PMSF, 1% protease inhibitor cocktail). After a 1.5-h incubation at 4 °C, the supernatant was removed with a 5-min spin at 1000 rpm, and the beads were washed twice with the lysis buffer at 4 °C. The recombinant GST-tagged proteins were eluted from the beads with the elution buffer (50 mM Tris pH 8.0, 10% glycerol, 0.3 M NaCl, 0.1% Triton X-100, 0.5 mM PMSF, 10 mM β-mercaptoethanol, 15 mM GSH).

The recombinant 6×His-tagged proteins were expressed in BL21 after induction with 0.2 mM IPTG at 16 °C and conjugated to Ni superflow beads in wash buffer (20 mM Hepes pH7.5, 10% glycerol, 1 M NaCl, 0.2% Triton X-100, 25 mM imidazole, 10 mM β-mercaptoethanol, 0.5 mM PMSF, 1% protease inhibitor cocktail). The beads were washed five times at 4 °C, and the recombinant 6×His-tagged proteins were then eluted from the beads with the elution buffer (20 mM Hepes pH7.5, 10% glycerol, 0.3 M NaCl, 0.35 M imidazole, 0.1% Triton X-100, 10 mM β-mercaptoethanol, 0.5 mM PMSF, 1% protease inhibitor cocktail).

### RNA isolation, reverse transcription, and quantitative real-time RT-PCR (qRT-PCR) analysis

Total RNAs were extracted using Trizol (Invitrogen, 15596018). cDNA synthesis was performed with 500 ng of total RNA using TransScript® All-in-One First-Strand cDNA Synthesis SuperMix (TransGen, AT341-01) according to the manufacturer’s instructions. mRNA levels were measured by qRT-PCR analysis based on SYBR® Premix Ex Taq™ (Takara, RR420A) with the BioRad real-time PCR machine. Results were normalized to β-actin. All the primers used in the study give rise to single product with the right size in agarose gel analysis. The data are presented as the mean ± SD (*t*-test; ***p < 0.001; **p < 0.01; *p < 0.05).

### Protein extraction and western blotting

Total protein was extracted by lysing cells with the whole-cell extraction buffer (50 mM Tris; 150 mM NaCl; 1% NP40; 10% glycerol; 1 mM EDTA; 1 mM PMSF). Twenty micrograms of the total proteins were separated by SDS–PAGE and transferred to PVDF membrane. The membrane was blocked with 5% milk and probed with specific primary antibodies and secondary antibodies. The blots were developed with ECL Advance Western Blotting Detection Kit (Amersham, #34080). The antibodies used in this study include anti-β-Catenin antibody (Cell signaling, #9587), anti-Ube2s antibody (Cell signaling, #11878s), anti-β-TrCP antibody (Cell signaling, #4394s), anti-Cdc27 antibody (Santa Cruz, sc-5618), anti-HA antibody (BETHYL, A-190-208A), anti-GST antibody (Santa Cruz, sc-138), anti-Ub (Cell signaling, #3936s), anti-P-β-Catenin T41/S45 (Cell signaling, #9565 s), anti-P-β-Catenin S33/S37/T41(Cell signaling, #9561s), anti-β-Tubulin (Santa Cruz, sc-166729), and anti-LaminB antibody (Santa Cruz, sc-6216).

### Immunoprecipitation

Five-hundred micrograms of protein samples in a total volume of 500 μl reaction solution were immunoprecipitated with 2 μg of antibody and 20 μl of Protein-G beads (GE Healthcare, 17-0618-01). The samples were rotated at 4 °C overnight. The beads were washed four times with 1 ml of cold NP40 lysis buffer containing protease inhibitors (Roche, 04693132001). The beads were then boiled for 10 min in the presence of 20 μl 2× sample buffer and the eluted proteins were fractionated by SDS-PAGE in 10% gels. Proteins were detected by immunoblotting as described above.

### GST pull-down assay

Purified GST-tagged protein were precleared with Glutathione Sepharose 4B (GE Healthcare, 17-0756-01) for 1.5 h and incubated with His-tagged fusion proteins at 4 °C overnight. Protein-bound sepharose beads were washed four times with lysis buffer and eluted in SDS-PAGE sample buffer. Eluted proteins were analyzed by immunoblotting.

### Immunocytochemistry

Cells were fixed with 4% formaldehyde for 30 min and washed four times with PBST buffer (0.25 g Tween-20 dissolved in PBS solution). Subsequently, the cells were blocked with 5% horse serum diluted in PBST buffer. Primary antibodies were applied in blocking solution at 4 °C overnight. The primary antibodies used in this study include anti-T antibody (Santa Cruz, sc-17745, 1:100) and anti-HA antibody (Santa Cruz, sc-7392, 1:500). After washing with PBST buffer, the coverslips were incubated with Alexa546-conjugated anti-mouse (Molecular Probes, 1:500) and Alexa488-conjugated anti-goat (Molecular Probes, 1:500) secondary antibodies for 1 h. The secondary antibody solution also contained 2 μg/ml DAPI. After washing with PBST buffer three times, the cell images were captured. All red and green images were adjusted identically in order to generate the merged images.

### In vitro ubiquitination assay

A 0.5-ml conjugation reaction containing 300 nM E1 (Uba1), 600 µM Ub, 500 nM Ube2s, 500 nM Ube2c, 5 µl Apc^Cdc27^ complex solution, and 200 nM β-Catenin in an ATP cocktail (10 mM Hepes, pH 7.5; 5 mM MgCl_2_; 5 mM ATP; 0.6 U/ml inorganic phosphatase) was incubated at 30 °C for 90 min. Reactions were terminated by addition of trichloroacetic acid (TCA) (final concentration of 10%), and subsequently analyzed by SDS-PAGE and western blotting with the antibody against β-Catenin (Cell signaling, #9587).

### Mesoendoderm differentiation assay

Mesoendoderm differentiation assay was performed as described previously by Thomson and colleagues^43^. Briefly, mES cell medium was changed to fresh N2B27 medium. After 48 h of culturing, 3 μM CHIR99021 (Sigma, SML1046-5MG) was added to treat the cells for 2 days. The resulting cells were analyzed by immunocytochemistry staining or real-time RT-PCR assays.

### Transwell assay

The transwell assay was conducted by using Transwells (Costar, Cambridge, MA, USA) with 8 μm pore-sized polycarbonate membrane filters in 24-well culture plates. The upper surface of the filter was coated with 12.5 μl Matrigel per filter (Becton Dickinson, Bedford, MA, USA). The Matrigel was dried and reconstituted at 37 °C into a solid gel on the filter surface. After starving in FBS-free DMEM overnight, 3 × 10^5^ cells were seeded in the upper chamber. The cells were allowed to invade for 48 h. Cells that invaded the lower surface of the filter were counted in five random fields under a light-microscope at high magnification.

### Scratch assay

The scratch assay was performed by seeding 2 × 10^5^ cells onto 96-well plates. Confluent monolayers were wounded using a pipette tip. The cell migration distance was measured under a light-microscope at high magnification in at least three random fields.

### Xenograft mouse model

The experimental mouse work followed the animal care protocol LA2013-1 approved by Peking University Animal Research Ethics Board and was conducted at the Peking University Health Science Center, China. For the in vivo tumorigenesis assay, 5 × 10^6^ cells from the *UBE2S*∆ cell line and the control line were injected subcutaneously into the bilateral flanks of 6-week-old nude mice, respectively. For the in vivo metastasis assay, the dox-inducible UBE2S overexpression HCT116 stable cell line was injected into tail veins of 6-week-old BALB/c female nude mice. For the group with UBE2S overexpression, dox was added in the feed after cell injection. The group without dox feed was used as control. Every group has seven mice. The mice were sacrificed 4 or 5 weeks after cell injection. Formalin-fixed lungs isolated were paraffin-embedded, and tissue sections were stained with H&E to evaluate the cellular morphology.

## Electronic supplementary material

Supplementary figure legends

Supplementary Figure S1

Supplementary Figure S2

Supplementary Figure S3

Supplementary Figure S4

Supplementary Figure S5
