# Supplementary material for: Ube2s stabilizes β-Catenin through K11-linked polyubiquitination to promote mesendoderm specification and colorectal cancer development
Source: Cell Death Dis. 2018 Apr 19;9(5):456. doi: 10.1038/s41419-018-0451-y (PMC5908793; doi:10.1038/s41419-018-0451-y)
Supplement: Supplementary file 1 — Supplementary figure legends [file 41419_2018_451_MOESM1_ESM.doc]

**Supplementary figure legends**

**Figure S1** Co-IP assay was used to detect the interaction between Ube2s and β-Catenin in F9 cells (a) and 293 cells (b). (c) Co-IP assay with the cytoplasm and nuclear extracts of mES cells was used to examine the Ube2s-β-Catenin complex formation, respectively. The data presented are based on three independent repeats.

**Figure S2** Ube2s promoted accumulation of β-Catenin. (a) *Ube2s* depletion decreased the protein level of β-Catenin in mES cells. Two constructs expressing *Ube2s* shRNAs were transfected into mES cells, respectively. Two days after transfection, whole cell extracts were obtained for western blotting assay with the antibodies against β-Catenin and Ube2s. (b) *UBE2S* knockdown reduced β-CATENIN in HCT116 cells. Two constructs expressing *UBE2S* shRNAs were transfected into HCT116 cells. Western blotting was used to monitor the protein level changes of β-CATENIN. β-TUBULIN serves as a loading control. (c) *UBE2S* overexpression induced β-CATENIN in HCT116 cells. The construct overexpressing *UBE2S* transfected into HCT116 cells. Similar experiment procedure was performed with (b). (d) Similar experiment with (c) was performed in HT29 cells. (e) Ube2s manipulation failed in significantly alerting the mRNA level of *β-Catenin* in mES cells. All data shown are based on three independent repeats.

**Figure S3** The regulatory activity of Ube2s toward β-Catenin. (a) co-IP to detect the association of β-Catenin with Cdc27. mES cell extracts were subjected to co-IP with the antibody against Cdc27, followed by western blotting with antibodies against β-Catenin. (b) UBE2S promoted β-CATENIN ubiquitination *in vivo* in HCT116 cells. Similar experimental procedure was employed with Figure 2d. (c) UBE2S elevation down-regulated repressive phospho-β-CATENIN in HCT116 cells. After two-day dox treatment, cytoplasm extracts of the HCT116 cell line expressing dox-inducible HA-UBE2S were prepared for western blotting assay with the antibodies against phospho-β-CATENIN. The untreated cells and wild-type (WT) HCT116 cells were used as controls. The antibody against TUBULIN was used as the cytoplasm marker. The data shown are based on three independent repeats.

**Figure S4** Ube2s overexpression stable mES cell line U2 was used to examine the role of Ube2s in promoting mES cell differentiation into mesoendoderm. (a) and (b) The U2 and control line (HA) were induced to differentiate into mesoendoderm. The resulting cells were analyzed by immunocytochemistry staining assay with antibodies against HA (Ube2s), T and DAPI (a) and qRT-PCR (b). Undifferentiated mES cells (wild-type, WT) were used as controls. Photographs were taken with 200x magnification. The mean DAPI-normalized staining signals were quantified by NIH Image J software. The values represent ratios against WT (a, the right panel). T-test: ***p < 0.001; **p < 0.01; *p < 0.05. The data presented are based on three independent repeats.

**Figure S5** Establishment of UBE2S∆ HCT116 cell line. (a)-(c) Schematic representation of target locus of endogenous UBE2S in the CRISPR/Cas9 system. Red letters indicate the insertion. Mutation of UBE2S was detected by PCR (b) and western blotting assay (c), respectively. (d) Western blotting assay were employed to check the expression of repressive phospho-β-CATENIN in UBE2S∆ HCT116 cells. (e) Western blotting assay were employed to check the expression of Wnt signaling target *CCND1* in UBE2S∆ HCT116 cells. (f) CHX treatment combining western blotting assay profiled β-CATENIN degradation in UBE2S∆-1 HCT116 cells.
