# Supplementary figures and images for: Ube2s stabilizes β-Catenin through K11-linked polyubiquitination to promote mesendoderm specification and colorectal cancer development
Source: Cell Death Dis. 2018 Apr 19;9(5):456. doi: 10.1038/s41419-018-0451-y (PMC5908793; doi:10.1038/s41419-018-0451-y)

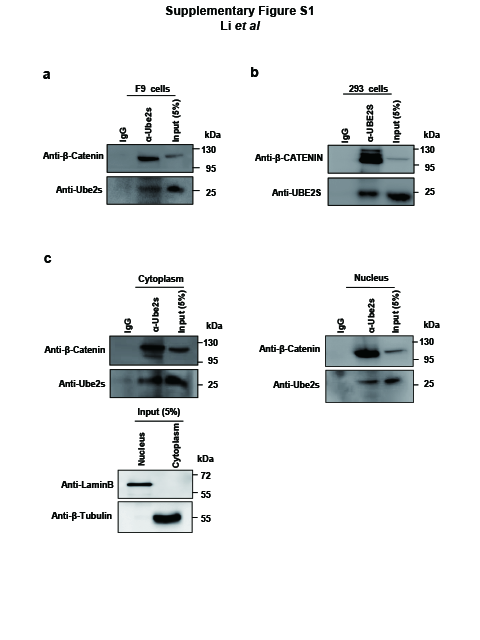

Supplement: Supplementary file 2 — Supplementary Figure S1 [file 41419_2018_451_MOESM2_ESM.tif]

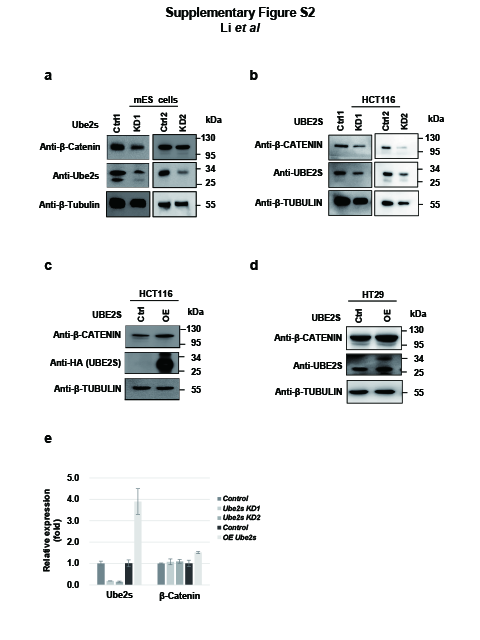

Supplement: Supplementary file 3 — Supplementary Figure S2 [file 41419_2018_451_MOESM3_ESM.tif]

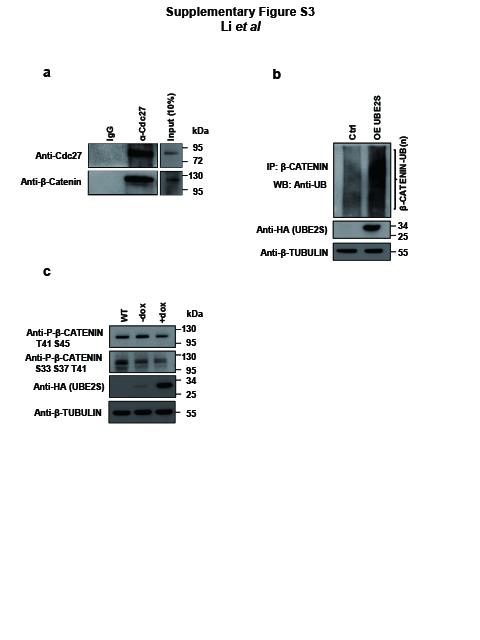

Supplement: Supplementary file 4 — Supplementary Figure S3 [file 41419_2018_451_MOESM4_ESM.tif]

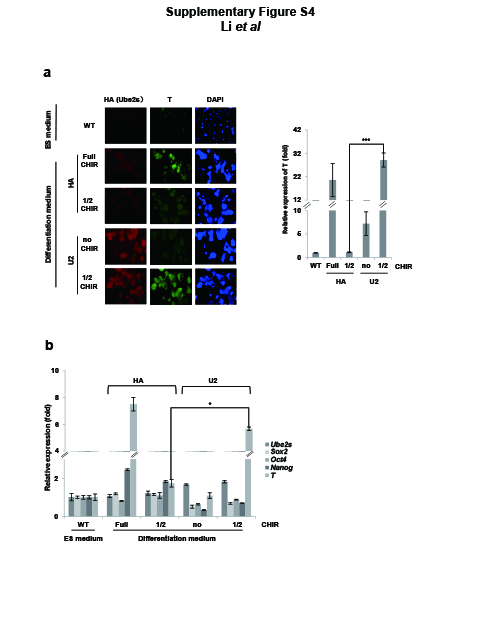

Supplement: Supplementary file 5 — Supplementary Figure S4 [file 41419_2018_451_MOESM5_ESM.tif]

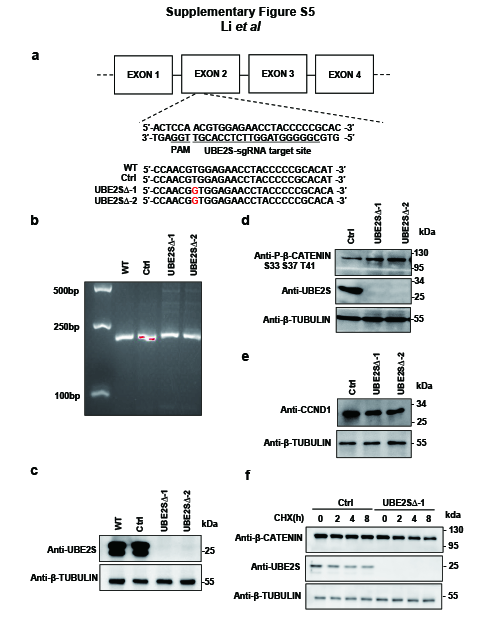

Supplement: Supplementary file 6 — Supplementary Figure S5 [file 41419_2018_451_MOESM6_ESM.tif]
